# Efficacy of dietary onion powder (Allium cepa L.) as a phytogenic feed additive: Effects on growth performance, blood biochemistry, gut morphology, and immune function in broiler chickens

**DOI:** 10.1016/j.psj.2026.107294

**Published:** 2026-06-13

**Authors:** Qiu Zhong, F. Raziq, S. Rahman, S. Khan, G. Abbas, M.T. Khan, R. Akhtar, E. Bughio, U. Farooq, Yue Yu, Z.M. Iqbal, Dalia Fouad, Yuwen Han

**Affiliations:** a Guangxi Key Laboratory of Animal Breeding, Disease Control and Prevention, College of Animal Science and Veterinary Medicine, Guangxi University, Nanning, Guangxi, China; b College of Animal Science and Technology, Guangxi Agricultural Vocational and Technical University, Nanning 530007, China; c Department of Poultry Production, Faculty of Animal Production and Technology, University of Veterinary and Animal Sciences, Lahore 54000, Pakistan; d Department of Poultry Science, Faculty of Animal Husbandry and Veterinary Sciences, The University of Agriculture, Peshawar 25130, Pakistan; e Department of Animal Production, Riphah College of Veterinary Sciences, Lahore 54000, Pakistan; f Department of Livestock and Poultry Sciences, Faculty of Veterinary and Animal Sciences, University of Layyah 31200, Pakistan; g Department of Pathology, Faculty of Veterinary Science, University of Veterinary and Animal Sciences, Lahore 54000, Pakistan; h Department of Poultry Production, Shaheed Benazir Bhutto University of Veterinary and Animal Sciences, Sakrand 67210, Pakistan; i Sub Campus, Toba Tek Sing, University of Agriculture, Faisalabad 36050, Pakistan; j College of Veterinary Medicine, Nanjing Agricultural University. No.1 Weigang, Xuanwu District, Nanjing 210095, China; k Department of Livestock Management, Faculty of Animal Production and Technology, Cholistan University of Veterinary and Animal Sciences, Bahawalpur 63100, Pakistan; l Department of Zoology, College of Science, King Saud University, PO Box 22452, Riyadh 11495, Saudi Arabia

**Keywords:** Onion powder, Growth performance, Gut health, Blood profile, Immune response

## Abstract

A study was carried out to evaluate the impact of dietary onion powder (OP) on growth performance, serum biochemistry, gut morphology, and immune response in broiler chickens. A total of 320 day-old chicks each with a uniform initial body weight of 42.50 ± 0.40 g were raised in an open-sided shed for six weeks using a completely randomized design. Birds were weighed and randomly divided into four treatment groups. Each group was replicated five times with 16 chicks each. The dietary treatments included: T₁ (basal diet), T₂ (OP at 3 g/kg feed), T_3_ (OP at 3.5 g/kg feed), and T_4_ (OP at 4 g/kg feed). Growth performance parameters, serum biochemical indices, intestinal histomorphology, and immune response traits were evaluated across all groups. Results demonstrated that OP supplementation significantly (*P=* 0.0001) improved growth performance. Cumulative feed intake and body weight gain increased in a dose-dependent manner, with the highest values recorded in the highest supplementation group (T_4_). Feed conversion ratio was also significantly improved, while mortality remained unaffected across all treatments (*P=* 0.640). Intestinal histomorphology was markedly enhanced, as OP increased villus height and villus height-to-crypt depth ratio, while reducing crypt depth, with the most pronounced effects observed in T_4_. Hematological indices, including red blood cells, white blood cells, hemoglobin, and packed cell volume, remained statistically unchanged (*P>* 0.05). Serum biochemical analysis revealed significant improvements (*P<* 0.05) in lipid and metabolic profiles, with reductions in cholesterol, triglycerides, glucose, and low-density lipoprotein, alongside increased high-density lipoprotein levels in OP-supplemented groups, particularly at higher OP-supplemented groups. Furthermore, humoral immune responses were significantly enhanced, with higher antibody titers against Newcastle Disease and Infectious Bronchitis. Dietary supplementation of onion powder, particularly at higher inclusion levels, effectively enhances growth performance, intestinal health, metabolic profile, and immune competence in broiler chickens without adversely affecting hematological stability. These findings suggest that onion powder can serve as a natural growth-promoting and health-enhancing feed additive in poultry production systems.

## Introduction

The global poultry industry is undergoing a paradigm shift toward antibiotic-free production systems, driven by growing concerns over antimicrobial resistance, drug residues in poultry products, and their associated public health implications (Abou-Jaoudeh et al., 2024). The ban on antibiotic growth promoters (**AGPs**), particularly in the European Union, has intensified the search for safe and effective alternatives capable of sustaining productivity while ensuring animal welfare and consumer safety (Michalczuk et al., 2024; Ibeagha-Awemu et al., 2025). In this regard, phytogenic feed additives (**PFAs**), derived from herbs, spices, and other plant-based sources, have emerged as promising candidates due to their diverse biological activities, including antimicrobial, antioxidant, and immunomodulatory properties (Wang et al., 2024). Accumulating evidence from high-impact studies demonstrates that PFAs can enhance feed efficiency, intestinal health, and immune competence in broiler chickens (Wang et al., 2021). The beneficial effects of PFAs are primarily attributed to their modulatory effects on gastrointestinal physiology and microbial ecology (Obianwuna et al., 2024; Oni and Oke, 2025). These bioactive compounds stimulate digestive enzyme secretion, improve nutrient digestibility, and promote a balanced gut microbiota by suppressing pathogenic bacteria while enhancing beneficial microbial populations (Biswas and Kim, 2025). Additionally, phytogenic compounds alleviate oxidative stress by strengthening endogenous antioxidant defense systems, thereby maintaining cellular integrity and optimizing physiological performance in poultry (Oke et al., 2024). Collectively, these multifunctional properties position PFAs as viable alternatives to synthetic growth promoters in modern broiler nutrition.

Among various phytogenic sources, onion (*Allium cepa* L.) has attracted considerable attention as a functional feed additive (Kothari et al., 2019). Onion is rich in bioactive compounds, including flavonoids (notably quercetin), organosulfur compounds, and phenolic acids, which exhibit potent antimicrobial, antioxidant, hypolipidemic, and immunostimulatory properties (Trigueros et al., 2024). These constituents play a crucial role in enhancing metabolic efficiency and maintaining physiological homeostasis in broiler chickens (Lee et al., 2025). Previous studies have indicated that dietary supplementation with onion powder (**OP**) or its extracts can improve growth performance, enhance feed conversion efficiency, and inhibit pathogenic microorganisms (Malematja et al., 2022). Additionally, onion supplementation has been shown to favorably modulate blood biochemical parameters by reducing serum cholesterol (**CH**) and triglyceride (**TR**) levels while improving protein metabolism (Malematja et al., 2023). These effects are largely attributed to the hypolipidemic and antioxidant properties of its phytochemicals, which regulate lipid metabolism and mitigate oxidative damage. Furthermore, onion-derived compounds have been reported to enhance immune function by stimulating antibody production and modulating cytokine responses, thereby improving resistance to infectious challenges (Cho et al., 2024). The positive effects of onion supplementation also extend to intestinal morphology, where improvements in villus height and epithelial turnover contribute to enhanced nutrient absorption and growth performance (Omar et al., 2020; Harman et al., 2024). In addition, the antimicrobial properties of onion help maintain gut health by limiting the proliferation of harmful microorganisms and reducing the incidence of enteric disorders (Obianwuna et al., 2024).

Despite its promising potential, variability in the reported efficacy of OP may arise due to differences in inclusion levels, processing techniques, and environmental or management conditions. Therefore, a comprehensive evaluation encompassing growth performance, blood biochemistry, gut morphology, and immune function is necessary to validate its practical applicability in broiler nutrition. In this context, the present study was designed to evaluate the efficacy of dietary OP (*Allium cepa* L.) as a phytogenic feed additive in broiler chickens. Specifically, the study aims to investigate its effects on growth performance, blood biochemical profile, intestinal morphology, and immune response, thereby contributing to the growing body of evidence supporting plant-based alternatives to AGPs in sustainable poultry production.

## Materials and methods

### Ethical note

All experimental procedures were conducted in accordance with ethical standards approved by Institutional Ethics Committee of the University of Agriculture, Peshawar (**UAP**) and adhered to national and institutional guidelines for the care and use of animals. The feeding trial lasted 42 days, which included a one-week adaptation period for the chicks prior to the start of the experiment.

### Experimental birds, design, and husbandry

A total of 320 day-old chicks each with a uniform initial body weight of 42.50 ± 0.40 g were raised in an open-sided shed for six weeks using a completely randomized design. They were weighed and randomly divided into four treatment groups. Each group was replicated five times with 16 chicks each (Table 1). The dietary treatments included: T₁ (basal diet), T₂ (OP at 3 g/kg feed), T_3_ (OP at 3.5 g/kg feed), and T_4_ (OP at 4 g/kg feed). The experiment was carried out in an open-sided poultry house measuring 30 ft × 40 ft × 10 ft, where 20 wire-mesh floor pens (3 ft × 3 ft × 3 ft) were used. Each pen was lined with a 3.5 cm layer of clean, dry wood shavings as bedding. Before the arrival of chicks, all pens, floors, and equipment were thoroughly cleaned and disinfected, and the facility was fumigated with formaldehyde five days in advance to ensure biosecurity. Brooding temperature was initially set at 32 ± 3°C during the first week and gradually decreased by 2°C per week until it reached 26°C in the fourth week, after which it was maintained for the rest of the trial. Relative humidity was maintained at 65 ± 5%. A continuous lighting program was applied for the first 24 hours, followed by a cycle of 23 hours light and 1 hour darkness throughout the experiment. Birds were provided ad libitum access to iso-caloric and iso-nitrogenous corn–soybean meal-based diets formulated according to the nutritional recommendations of Ross-308 broilers (Aviagen, 2016), presented in mash form (Table 2). Feed and water were available at all times. Each pen was equipped with two feeders and one nipple drinker, and their height was adjusted progressively in accordance with bird growth and age.

**Table 1:** Experimental design.

Group	Treatment^1^	No. of birds
T_1_	Basal diet without supplements (control)	80
T_2_	Basal diet with OP at 3g/k of feed	80
T_3_	Basal diet with OP at 3.5g/k of feed	80
T_4_	Basal diet with OP at 4g/kg of feed	80

1 OP: onion powder.

**Table 2:** Composition of the basal diet for starter and finisher phases.

Ingredients (%)	Starter phase (1–21 days)	Finisher phase (22–42 days)
Maize	53.50	58.19
Soybean meal (44% CP)	36.00	31.00
Fish meal (55% CP)	3.00	2.00
Vegetable oil	3.50	5.00
Dicalcium phosphate	1.80	1.75
Limestone	1.10	1.00
Sodium chloride	0.30	0.30
DL-Methionine	0.13	0.12
Threonine	0.07	0.04
Sodium bicarbonate	0.10	0.10
Vitamin premix^1^	0.20	0.20
Minerals premix^2^	0.30	0.30
Total	100	100
Nutrient		
Metabolizable energy (kcal/kg)	3000	3150
Crude protein	22.00	20.00
Calcium	0.90	0.88
Available phosphorus	0.45	0.40
Lysine	1.20	1.10
Methionine	0.50	0.45

1 Provided per kg of diet: vitamin A, 11,000 IU; vitamin D_3_, 2,560 IU; vitamin E, 44 IU; vitamin K, 4.2 mg; riboflavin, 8.5 mg; niacin, 48.5 mg; thiamine, 3.5 mg; d-pantothenic, 27 mg; choline, 150 mg; vitamin B_12_, 33 μg.

2 Provided per kg of diet: manganese, 60 mg; zinc, 70 mg; iron, 30 mg; copper, 10 mg; iodine, 0.45 mg; selenium, 0.2 mg.

### Collection, processing, and phytochemical analysis of onion bulb

Fresh onion bulbs (*Allium cepa*) were procured from a local market, thoroughly cleaned, and sliced into thin sections. The slices were air-dried under ambient conditions (34 ± 2°C) for six days until a constant weight was achieved, and were subsequently ground into a fine powder using an electric grinder. The resulting OP was subsequently used for chemical analyses. The proximate composition of the raw onion was determined according to the standard methods of the AOAC (2000), and the results are presented in Table 3. Phytochemical characterization of the OP was performed following the methods described by Samatha et al. (2012) and Oluwaniyi et al. (2014). Total phenolic content was measured using the Folin–Cicocalteau colorimetric assay with gallic acid as the reference standard, and the results were expressed as milligrams of gallic acid equivalent (**GAE**) per kilogram of dry weight (**DW**). Total flavonoid content was determined using the aluminum chloride colorimetric method, employing quercetin as the calibration standard, and the values were reported as milligrams of quercetin equivalents (**QE**) per kilogram DW. The phytochemical composition of the OP is presented in Table 4.

**Table 3:** Proximate composition of raw onion bulbs.

Nutrient	Composition
Moisture (%)	89.31
Crude protein (%)	1.42
Lipids (%)	0.15
Ash (%)	0.39
Carbohydrates (%)	8.12
Crude fiber (%)	1.94
Energy (kcal)	39.55

**Table 4:** Phytochemical analysis of onion powder.

Components (%)	Quantity
Total flavonoid (mg QE/kg DW)	4250
Total phenolic (mg GAE/kg DW)	2800
Tannin (mg/kg DW)	8.0
Saponin (mg/kg DW)	7.7
Anthocyanin (mg/kg DW)	0.80
Alkaloid (mg/kg DW)	8.1
Terpenoids (mg/kg DW)	1.8

DW: dry weight, QE: quercetin equivalent, GAE: gallic acid equivalent.

### Data collection

#### Growth performance and gut morphology

Data was recorded for body weight gain (**BWG**) on weekly basis. Chicks were weighed at day first and then at the end of each week. Initial weight was subtracted from final weight to obtain weight gain. Total weight gain was calculated at the end of study. Feed was offered to each group ad-libitum on daily basis, and the refusal was weighed at next morning and subtracted from the daily feed offered to calculate daily feed intake. The total feed intake was calculated at the end of study. Feed conversion ratio (**FCR**) was calculated on weekly basis by dividing weekly feed intake on weekly weight gain basis. Total FCR was calculated from the total feed intake divided by weight gain at the end of trial. At the end of the experimental period, four birds from each replicate were randomly selected and slaughtered and gut morphology parameters, including villus height (**VH**), crypt depth (**CD**), and the villus height-to-crypt depth ratio (**VH:CD**), were measured following the method described by Li et al. (2026).

#### Hematobiochemical profile and immune response

At the time of slaughter, blood samples (approximately 3 mL) were obtained from four birds per replicate and transferred into EDTA-coated tubes for hematological evaluation. The samples were analyzed for red blood cell (**RBC**) count, white blood cell (**WBC**) count, packed cell volume (**PCV**), and hemoglobin (**Hb**) concentration using an automated hematology analyzer (Cell-DYN 3500; Abbott Diagnostics, USA). For serum biochemical analysis, another portion of blood was collected in non-heparinized tubes, allowed to clot, and then centrifuged at 4,000 × g for 15 minutes at 4°C to separate the serum. The harvested serum was subsequently stored at −20°C until further analysis. Biochemical indices, including total CH, glucose (**GL**), TR, low-density lipoprotein (**LDL**), and high-density lipoprotein (**HDL**), were determined from the serum samples. In addition, antibody titers against Newcastle disease (**ND**) infectious bronchitis (**IB**) viruses were evaluated using the hemagglutination inhibition (HI) test and enzyme-linked immunosorbent assay (ELISA) with commercial diagnostic kits (BioChek, Gouda, the Netherlands). All birds had previously been vaccinated against ND and IB using live attenuated vaccines following the manufacturer’s recommendations, with vaccination administered one week prior to blood sampling.

#### Statistical analysis

Data were subjected to statistical evaluation using a one-way analysis of variance (ANOVA) within a completely randomized design (CRD) framework, performed with SAS software (SAS Institute Inc, 2002). When significant differences were detected, treatment means were separated using Duncan’s Multiple Range Test at a probability level of *P* *<* 0.05. The experimental unit for all analyses was the individual pen. The following statistical model was applied:

Yij = µ + τi + εij

Where: Yij = Observed dependent variable; μ = Overall mean; τi = Effect of the ith treatment; and εij = Residual error.

## Results

### Growth performance

Dietary supplementation of OP significantly (*P* *=* 0.0001) enhanced the growth performance of broiler chickens at 42 days of age (Table 5). Cumulative feed intake (**CFI**) increased significantly in all supplemented groups, reaching the highest value in T_4_, followed by T_3_ and T_2_, whereas the control group (T_1_) exhibited the lowest intake. A similar trend was observed for BWG, with T_4_ recording the greatest gain, indicating a clear dose-dependent response. Feed conversion ratio was significantly enhanced by onion supplementation, with T_4_ showing the best efficiency compared to the control, while T_2_ and T_3_ exhibited intermediate values. However, mortality was not significantly affected (*P* *=* 0.640), although a slight numerical decline was observed in the supplemented groups.

**Table 5:** Effects of dietary onion powder supplementation on the growth performance of broiler chickens at 42 days of age.

Treatments^2^	Parameters^1^			
	CFI (g)	BWG (g)	FCR	M (%)
T_1_	2922^d^±7.06	1831^d^±3.02	1.60^a^±0.02	1.50±0.64
T_2_	3130^bc^±7.34	1985^bc^±5.06	1.58^ab^±0.07	1.35±0.48
T_3_	3156^b^±6.97	2011^b^±5.97	1.57^ab^±0.04	1.15±0.29
T_4_	3180^a^±5.03	2041^a^±4.03	1.56^b^±0.02	1.15±0.25
P-value	0.0001	0.0001	0.0001	0.640

a-d Different superscripts on means within a column show significant difference (P < 0.05).

1 CFI: cumulative feed intake, BWG: body weight gain, FCR: feed conversion ratio, M: mortality.

2 T1: basal diet (control); T2: basal diet with onion powder at 3g/kg of feed; T3: basal diet with onion powder at 3.5g/kg of feed; T4: basal diet with onion powder at 4g/kg of feed.

### Gut morphology

Dietary supplementation of OP significantly (*P* *=* 0.0001) enhanced intestinal histomorphology in broiler chickens (Table 6). Villus height increased progressively with increasing inclusion levels, with the highest value recorded in T_4_ and the lowest in the control group (T_1_). In contrast, CD decreased significantly, ranging from the highest value in T_1_ to the lowest in T_4_. As a result, the VH:CD was significantly improved, with T_4_ showing the highest ratio and T_1_ the lowest.

**Table 6:** Effects of dietary onion powder supplementation on intestinal histomorphology of broiler chickens.

Treatments^2^	Parameters^1^		
	VH (µm)	CD (µm)	VH:CD
T_1_	1540^d^±16.12	225^a^±6.92	6.84^d^±0.91
T_2_	1570^c^±20.74	210^b^±4.08	7.48^c^±1.02
T_3_	1595^b^±22.46	190^c^±4.12	8.40^b^±1.10
T_4_	1630^a^±18.62	178^d^±3.74	9.16^a^±0.88
P-value	0.0001	0.0001	0.0001

a-d Different superscripts on means within a column show significant difference (P < 0.05).

1 VH: villus height, CD: crypt depth.

2 T1: basal diet (control); T2: basal diet with onion powder at 3g/kg of feed; T3: basal diet with onion powder at 3.5g/kg of feed; T4: basal diet with onion powder at 4g/kg of feed.

### Hematological parameters

Dietary supplementation of OP had no significant effect (*P* *>* 0.05) on the hematological parameters of broiler chickens (Table 7). Red blood cell count, hemoglobin concentration, and packed cell volume remained statistically comparable across all treatment groups. Similarly, white blood cell count was not significantly affected, although a slight numerical increase was observed with increasing inclusion levels.

**Table 7:** Effects of dietary onion powder supplementation on hematological parameters of broiler chickens at 42 days of age.

Treatments^2^	Parameters^1^			
	RBC × 10^6^	WBC × 10^3^	Hb (mg/dL)	PCV (%)
T_1_	3.000±0.11	142±3.20	7.70±0.20	24.8 ± 0.93
T_2_	3.001±0.25	144±2.18	7.85±0.06	25.2 ± 1.15
T_3_	3.000±0.30	145±3.08	7.75±0.22	24.8 ± 0.91
T_4_	3.002±0.28	148±2.10	7.77±0.20	25.3 ± 0.91
P-value	0.418	0.463	0.590	0.751

Same superscripts on means within a column show significant difference (*P<* 0.05).

1 RBC: red blood cells, WBC: white blood cells, Hb: hemoglobin, PCV: packed cell volume.

2 T_1_: basal diet (control); T_2_: basal diet with onion powder at 3g/kg of feed; T_3_: basal diet with onion powder at 3.5g/kg of feed; T_4_: basal diet with onion powder at 4g/kg of feed.

### Serum biochemical parameters

Dietary supplementation of OP significantly (*P* *<* 0.05) improved serum biochemical profile of broiler chickens (Table 8). Cholesterol, glucose, triglycerides, and LDL levels decreased progressively with increasing inclusion levels, reaching their lowest values in T_4_ compared with the highest values observed in the control group (T_1_). In contrast, HDL concentration increased significantly in T_4_ compared to T_1_ group, indicating an improved lipid profile.

**Table 8:** Effect of dietary onion powder supplementation on serum biochemistry of broiler chickens at 42 days of age.

Treatments^2^	Parameters^1^				
	CH (mg/dL)	GL (mg/dL)	TR (mg/dL)	HDL (mg/dL)	LDL (mg/dL)
T_1_	146^a^±2.26	80.5^a^±1.94	130^a^±2.08	38.0^d^±1.29	123^a^±3.48
T_2_	135^b^±2.60	70.1^b^±1.34	120^ab^±2.07	43.5^c^±2.75	120^b^±3.07
T_3_	132^c^±2.18	65.8^c^±1.09	118^b^±2.09	45.3^b^±2.66	113^c^±3.19
T_4_	128^d^±2.98	64.7^d^±1.96	111^c^±2.17	53^a^±2.88	110^d^±2.50
P-value	0.001	0.0001	0.011	0.001	0.021

a-d Different superscripts on means within a column show significant difference (P < 0.05).

1 CH: cholesterol, GL: glucose, TR: triglyceride, HDL: high-density lipoprotein, LDL: low-density lipoprotein.

2 T1: basal diet (control); T2: basal diet with onion powder at 3g/kg of feed; T3: basal diet with onion powder at 3.5g/kg of feed; T4: basal diet with onion powder at 4g/kg of feed.

### Immune antibody response

Dietary supplementation of OP significantly enhanced humoral immune responses in broiler chickens (Table 9), as evidenced by increased antibody titers against ND (*P* *=* 0.014) and IB (*P* *=* 0.025). ND HI titers increased in a dose-dependent manner, with the highest value recorded in T_4_ and the lowest in the control group (T_1_). A similar trend was observed for IB ELISA titers, which increased progressively with supplementation, reaching the highest level in T_4_ compared to T_1_.

**Table 9:** Effect of dietary onion powder supplementation on immune response of broiler chickens.

Treatments^2^	Antibody titers^1^	
	ND (HI titer, log_2_)	IB (ELISA titer)
T_1_	5.10^b^±0.76	3346^b^±10.54
T_2_	5.11^b^±0.48	3390^b^±9.22
T_3_	5.15^ab^±0.88	3470^ab^±11.88
T_4_	5.18^a^±0.62	3592^a^±11.18
P-value	0.014	0.025

a-b Different superscripts on means within a column show significant difference (P < 0.05).

1 Birds were vaccinated via drinking water using commercially available ND (La Sota) and IB (H 120) vaccines, one week before blood samples were taken.

2 T1: basal diet (control); T2: basal diet with onion powder at 3g/kg of feed; T3: basal diet with onion powder at 3.5g/kg of feed; T4: basal diet with onion powder at 4g/kg of feed.

## Discussion

### Growth performance

Measuring growth performance parameters in broiler chickens following dietary intervention is essential because they directly reflect the effectiveness of the diet in supporting nutrient utilization, metabolism, and overall productivity. Parameters such as body weight gain, feed intake, and feed conversion ratio offer a practical and economically relevant evaluation of how dietary treatments influence growth efficiency and biological productivity in poultry production systems (Torrey et al., 2021; Quintana-Ospina et al., 2023). Dietary supplementation of OP significantly improved growth performance in broiler chickens, as evidenced by increased CFI, higher BWG, and improved FCR, with the greatest response observed at 4 g/kg (T_4_), indicating a clear dose-dependent effect. These improvements can be attributed to the onion-derived bioactive compounds, including organosulfur compounds, flavonoids (e.g., quercetin), and prebiotic fructooligosaccharides. These constituents enhance feed palatability and stimulate digestive secretions, thereby increasing feed intake, while simultaneously modulating gut microbiota by promoting beneficial bacteria and suppressing pathogenic populations (Abdel-Latif et al., 2021; Sun et al., 2022). This microbial balance, along with enhanced intestinal morphology and increased digestive enzyme activity, further promotes nutrient digestibility and absorption, leading to greater weight gain and improved feed efficiency. Furthermore, the strong antioxidant and anti-inflammatory properties of onion constituents mitigate oxidative stress and support metabolic functions, contributing to improved growth performance (Dong et al., 2020; Wang et al., 2022). The lack of significant effect on mortality that, under optimal rearing conditions, the benefits of OP are primarily expressed through enhanced physiological efficiency rather than survival.

These findings are consistent with recent studies demonstrating that onion supplementation improves growth performance, gut health, and nutrient utilization in poultry. For instance, phenolic-rich onion extract has been shown to enhance growth performance, intestinal histology, and antioxidant activity in broilers (Omar et al., 2020), while onion extract inclusion improved growth traits and carcass characteristics (Malematja et al., 2023). Similarly, recent work using onion peel powder reported improvements in growth performance and physiological parameters in broilers (Alamuoye et al., 2024). In addition, studies on Allium-based additives confirm their role in improving intestinal morphology and growth efficiency through modulation of gut microbiota and antioxidant mechanisms (Adeyemi et al., 2021), while recent experimental evidence with onion and garlic powders further supports improvements in performance and intestinal health in poultry (Harman et al., 2024). Overall, these findings substantiate the efficacy of OP at 4 g/kg as a potent natural growth promoter in broiler production systems.

### Gut morphology

Assessing intestinal morphology parameters in broiler chickens after dietary intervention is important as it offers a direct evaluation of gut health, particularly regarding the structural organization and absorptive efficiency of the intestine. Key indicators such as VH, CD, and the VH:CD ratio are widely used to determine digestive and absorptive capacity, providing valuable information on how different diets affect intestinal development and overall physiological function. Dietary supplementation of OP significantly improved intestinal histomorphology in broiler chickens, as evidenced by increased VH, reduced CD, and a higher VH:CD ratio, with the greatest response observed at 4 g/kg (T_4_), indicating a clear dose-dependent effect. Mechanistically, these improvements are attributed to the bioactive constituents of onion, including organosulfur compounds (e.g., thiosulfinates), flavonoids (notably quercetin), and prebiotic fructooligosaccharides. Collectively, these compounds enhance gut health by suppressing pathogenic bacteria and promoting beneficial microbiota, thereby reducing intestinal inflammation and epithelial turnover (Dong et al., 2020; Abdel-Latif et al., 2021; Sun et al., 2022). The consequent reduction in enterocyte loss lowers crypt depth, while enhanced proliferation and differentiation of intestinal epithelial cells promote villus elongation, increasing the absorptive surface area. In addition, fermentation of prebiotic components stimulates the production of short-chain fatty acids, particularly butyrate, which serve as a key energy source for enterocytes and promotes mucosal development and integrity (Liu et al., 2021; Mallo et al., 2021). The potent antioxidant properties of onion bioactives further protect intestinal tissues from oxidative damage, preserving villus structure and function (Dong et al., 2020; Sun et al., 2020). Thus, the improved VH:CD ratio reflects enhanced digestive efficiency and nutrient absorption capacity.

These findings are strongly supported by recent studies. Omar et al. (2020) and Gazwi et al. (2022) reported that phenolic-rich onion extract significantly enhanced villus height, mucosal thickness, and overall intestinal development in broilers, indicating improved absorptive capacity. Similarly, Malematja et al. (2023) observed increased villus height and improved nutrient absorption in broilers supplemented with onion extract. Adeyemi et al. (2021) also observed enhanced intestinal development and reduced crypt depth with polyphenol-rich phytogenic additives. More recently, Alamuoye et al. (2024) showed that onion peel powder significantly improved villus height and gut health parameters. In addition, Harman et al. (2024) reported improved intestinal integrity and villus architecture following Allium-based supplementation. Comparable findings were reported by Surasorn et al. (2024), who demonstrated significant improvements in VH, CD, and VH:CD ratio using shallot-derived additives, highlighting the conserved gut-modulatory effects of Allium species. Furthermore, Dokou et al. (2023) confirmed that phytogenic feed additives consistently enhance villus height and VH:CD ratio while reducing crypt depth, reflecting improved intestinal functionality and reduced maintenance energy demands. Overall, the present findings clearly demonstrate that OP supplementation, particularly at 4 g/kg, optimizes intestinal architecture by promoting villus development, reducing crypt activity, and enhancing mucosal integrity, thereby maximizing nutrient absorption and contributing to improved growth performance in broiler chickens.

### Hematological parameters

Dietary supplementation of OP up to 4 g/kg had no significant effect on hematological parameters of broiler chickens, as indicated by comparable values of RBC count, Hb, PCV, and WBC across all treatments. This stability indicates that OP, at the tested inclusion levels, does not interfere with erythropoiesis, oxygen transport capacity, or immune cell homeostasis, thereby maintaining normal physiological status. Onion-derived bioactive compounds—particularly organosulfur compounds and flavonoids such as quercetin—exert predominantly protective effects under non-stress conditions. Their strong antioxidant activity maintains redox balance by scavenging reactive oxygen species, thereby preventing oxidative damage to erythrocyte membranes and hemoglobin (Sierżant et al., 2023), which helps stabilize RBC indices rather than elevating them (Remigante et al., 2022a,b; Wu et al., 2022). Likewise, the absence of significant changes in WBC count suggests that OP did not induce systemic immune activation or inflammation, while the slight numerical increase may reflect mild immunomodulatory effects associated with improved gut health and microbial balance.

Furthermore, hematological parameters are relatively stable indicators of systemic health and typically respond only under conditions of stress, toxicity, or disease challenge (Nwaigwe et al., 2020). The unchanged Hb and PCV values indicate that iron metabolism and nutrient assimilation were not compromised, whereas the stable RBC count confirms the absence of hemolytic or toxic effects. This further supports the biological safety of OP supplementation and suggests that its positive effects on growth performance and intestinal morphology occur without imposing physiological stress or disrupting blood homeostasis.

These results are in agreement with recent studies reporting no significant alterations in hematological indices following supplementation with onion or other Allium-derived phytogenics. Omar et al. (2020) and Malematja et al. (2023) both reported unchanged RBC, Hb, and PCV values in broilers supplemented with onion extracts, despite improvements in performance and gut health. Similarly, Adeyemi et al. (2021) observed stable hematological indices in broilers fed polyphenol-rich phytogenic additives, indicating no adverse systemic effects. Alamuoye et al. (2024) further confirmed that onion peel powder did not significantly affect hematological traits, reinforcing its safety in poultry diets. Comparable results have been reported with other Allium species, where improvements in performance and intestinal health occurred without significant changes in blood parameters (Harman et al., 2024; Surasorn et al., 2024). In addition, broader evidence suggests that phytogenic feed additives generally exert stabilizing rather than stimulatory effects on hematological variables under normal rearing conditions (Dokou et al., 2023). Overall, the present results clearly demonstrate that OP supplementation up to 4 g/kg is physiologically safe and does not adversely affect hematological indices in broiler chickens while supporting improved growth performance and gut health in broiler chickens.

### Serum biochemical parameters

Measuring serum biochemical parameters in broiler chickens following dietary intervention is critical for evaluating metabolic health, nutrient utilization efficiency, and overall physiological status (Amer et al., 2021). Key indicators such as glucose, cholesterol, triglycerides, and protein fractions provide valuable insights into how dietary components influence energy metabolism, lipid regulation, and organ function. Additionally, they help assess the safety and efficacy of feed additives by detecting potential metabolic imbalances or health improvements (Omar et al., 2021; Zálešáková et al., 2025). In the present study, dietary supplementation of OP significantly improved the serum biochemical profile of broiler chickens, as evidenced by progressive reductions in CH, TR, L, and LDL levels, alongside a marked increase in HDL concentration, with the most favorable response observed at 4 g/kg (T_4_), indicating a clear dose-dependent effect. These improvements can be attributed to the hypolipidemic, hypoglycemic, and antioxidant properties of onion-derived bioactive compounds, particularly organosulfur compounds (e.g., S-alk(en)yl cysteine sulfoxides), flavonoids such as quercetin, and phenolic acids. These compounds inhibit key lipogenic enzymes, such as HMG-CoA reductase, thereby suppressing endogenous cholesterol synthesis, while enhancing bile acid excretion to facilitate cholesterol clearance (Mahdavi et al., 2020; Elattar et al., 2024). Additionally, onion bioactives upregulate lipoprotein lipase activity and improve hepatic lipid metabolism, leading to reduced triglyceride and LDL concentrations and a concomitant increase in HDL levels (Huang et al., 2021; Fogacci et al., 2024). The observed reduction in blood glucose may be linked to improved insulin sensitivity, modulation of carbohydrate-metabolizing enzymes, and reduced intestinal glucose absorption, partly mediated by the prebiotic components of onion.

Furthermore, the strong antioxidant capacity of quercetin and related compounds reduces oxidative stress and lipid peroxidation, thereby protecting hepatic function and improving overall metabolic efficiency (Zhao et al., 2021; Qi et al., 2022; Shabir et al., 2022). The prebiotic fraction of onion further modulates gut microbiota, promoting the production of short-chain fatty acids (particularly butyrate), which play a key role in regulating lipid and glucose metabolism at the systemic level. Collectively, these mechanisms explain the improved serum biochemical profile and indicate enhanced metabolic health in OP-supplemented birds.

These findings are well supported by recent literature. Omar et al. (2020) reported significant reductions in serum cholesterol and triglycerides, along with improved antioxidant status, in broilers supplemented with onion extract. Malematja et al. (2023) similarly observed decreased total CH and LDL, accompanied by improved lipid metabolism. Adeyemi et al. (2021) demonstrated that polyphenol-rich phytogenic additives enhance lipid profiles and reduce oxidative stress in poultry. Alamuoye et al. (2024) further confirmed reductions in serum cholesterol, triglycerides, and glucose in broilers fed onion peel powder. In addition, Harman et al. (2024) reported improved lipid metabolism and reduced serum cholesterol with Allium-based supplementation, while Surasorn et al. (2024) documented decreased triglycerides and LDL alongside increased HDL in broilers supplemented with shallot powder. Dokou et al. (2023) also highlighted the consistent role of phytogenic feed additives in improving lipid metabolism and antioxidant status in poultry. Overall, the present findings clearly demonstrate that OP supplementation, particularly at 4 g/kg, effectively regulates lipid and glucose metabolism, enhances antioxidant status, and improves overall metabolic health in broiler chickens, supporting its use as a safe and effective natural alternative to conventional growth promoters.

### Immune antibody response

Measuring antibody-mediated immune responses (e.g., ND HI and IB ELISA titers) in broiler chickens is a critical tool to evaluate the efficacy of dietary interventions, as it directly reflects the functional competence of the humoral immune system and the bird’s ability to respond to vaccination (Behboodi et al., 2021; Teng et al., 2021). Enhanced antibody titers indicate improved immunocompetence, disease resistance, and overall health status, thereby serving as a reliable biomarker for assessing the immunomodulatory potential of feed additives (Naveed et al., 2020; Ike et al., 2021; Shi et al., 2024; Haque et al., 2024). The present study demonstrated that dietary supplementation of OP significantly enhanced humoral immune responses in broiler chickens, as evidenced by increased antibody titers against ND and IB. Notably, both ND hemagglutination inhibition (HI) and IB ELISA titers exhibited a clear dose-dependent increase, with the highest values observed in birds receiving 4 g/kg OP (T_4_). This finding suggests that OP acts as a potent natural immunomodulator, capable of improving vaccine-induced immune responses in poultry. The immunostimulatory effects observed in the present study can be primarily attributed to the rich profile of bioactive compounds present in onion (*Allium cepa*), including flavonoids (particularly quercetin), phenolic acids, and organosulfur compounds. These bioactives are known to enhance immune competence by stimulating B-lymphocyte proliferation and differentiation, thereby promoting immunoglobulin synthesis (Yang et al., 2020; El-Kazaz et al., 2024). Furthermore, they modulate cytokine production, particularly interleukins involved in humoral immunity, which ultimately leads to improved antibody titers following vaccination (Xu et al., 2024). Similar findings have been reported by Adeyemi et al. (2021), who observed elevated immunoglobulin levels and enhanced immune responsiveness in broilers supplemented with onion-derived products. Likewise, Omar et al. (2020) demonstrated that phenolic-rich onion extracts significantly improved IgG concentrations and immune status in poultry.

Another plausible mechanism underlying the enhanced antibody response is the stimulatory effect of onion bioactives on lymphoid organ development. The bursa of Fabricius, spleen, and thymus play central roles in the maturation and proliferation of immune cells (Song et al., 2021; Lee et al., 2024; Amaz et al., 2025). Dietary onion supplementation has been reported to promote lymphoid organ growth and functionality, particularly by increasing the relative weights of the thymus and bursa of Fabricius and enhancing immunoglobulin production, thereby increasing the population of antibody-producing cells and improving overall humoral immune responses in broiler chickens (Omar et al., 2020; Noruzi and Aziz‐Aliabadi, 2024). This enhancement in lymphoid tissue activity likely contributed to the improved ND and IB titers observed in the present study. Supporting this notion, previous research has indicated that phytogenic compounds have been shown to enhance lymphoid organ indices and stimulate immune cell proliferation, leading to improved humoral immune responses and increased antibody production in poultry (Abdelli et al., 2021; Ogwuegbu and Mthiyane, 2024; Ntsongota et al., 2025).

In addition, the antioxidant properties of onion may have played a critical role in enhancing immune responses. Oxidative stress is a well-known suppressor of immune function, as excessive production of reactive oxygen species disrupts immune homeostasis, impairs lymphocyte activity, and diminishes antibody production, thereby increasing susceptibility to infections and reducing overall immunocompetence in poultry (Li et al., 2024; Oke et al., 2024). Onion is a rich source of natural antioxidants, particularly quercetin, which effectively scavenges reactive oxygen species and protects immune cells from oxidative damage (Dong et al., 2020; Marefati et al., 2021; Savitha et al., 2021). By maintaining cellular integrity and improving redox balance through activation of antioxidant defense systems and reduction of oxidative damage, onion supplementation enhances the efficiency of immune responses, particularly under immunological challenges such as vaccination (Dong et al., 2020; Sun et al., 2020). Consistent with these findings, recent studies have reported that dietary supplementation of onion and its derivatives significantly improves antioxidant status by enhancing endogenous antioxidant enzyme activity, while simultaneously promoting immune function through increased immunoglobulin production and immune cell activity in broiler chickens (Omar et al., 2020; Dosoky et al., 2021; Sravani et al., 2023).

Furthermore, the improvement in humoral immunity may also be linked to the modulatory effects of onion on gut microbiota. Onion contains prebiotic compounds, particularly fructooligosaccharides and related non-digestible carbohydrates, which selectively stimulate the growth of beneficial bacteria such as *Lactobacillus* and *Bifidobacterium*, while simultaneously suppressing pathogenic microorganisms. This favorable shift in gut microbial composition enhances intestinal integrity and stimulates metabolic outputs such as short-chain fatty acids, which are closely linked to improved immune function through the gut-associated lymphoid tissue (Lu et al., 2021; Yoo et al., 2025; Ebaid et al., 2026). Enhanced nutrient absorption and antigen presentation further contribute to a more robust immune response. Adeyemi et al. (2021) also reported that onion supplementation improved gut microbial balance and immune performance in broilers, supporting the gut–immune axis hypothesis.

The dose-dependent response observed in the present study, with T_4_ (4 g/kg) yielding the most pronounced improvement in ND and IB titers, suggests that higher inclusion levels of OP provide greater concentrations of bioactive compounds, thereby exerting stronger immunostimulatory effects. This trend is consistent with the findings of Noruzi and Aziz‐Aliabadi (2024), who reported a linear improvement in antibody titers with increasing levels of phytogenic feed additives. Similarly, recent work by demonstrated enhanced immune responses in broilers supplemented with onion, particularly at higher inclusion rates. Recent advances also indicate that phytogenic additives can modulate immune-related gene expression, further strengthening both innate and adaptive immunity (Ahmad et al., 2024).

### Conclusions

The present study demonstrates that dietary supplementation with OP significantly enhances growth performance, intestinal morphology, serum biochemical profiles, and humoral immune responses in broiler chickens, without adversely affecting mortality or hematological parameters. Birds supplemented with OP, particularly at 4 g/kg of feed, exhibited improved feed intake, body weight gain, feed conversion efficiency, along with substantial improvements in intestinal health, as evidenced by increased villus height and villus height-to-crypt depth ratio. Furthermore, OP supplementation strengthened immune competence by significantly increasing antibody titers against Newcastle Disease and Infectious Bronchitis. In addition, OP favorably modulated lipid metabolism by reducing serum cholesterol, triglycerides, glucose, and LDL levels while elevating HDL concentration, indicating enhanced metabolic health. The absence of adverse effects on hematological indices further confirms the physiological safety of OP supplementation in broiler diets. Collectively, these findings suggest that dried onion powder can serve as an effective natural feed additive, capable of enhancing growth performance, intestinal health, metabolic efficiency, and immune competence in broiler chickens. Its incorporation into poultry diets may provide a sustainable and effective alternative to conventional synthetic growth promoters, thereby supporting the increasing global demand for antibiotic-free and organic poultry production systems. However, further large-scale studies under diverse management and environmental conditions are warranted to validate these findings and establish the most effective inclusion rates for practical industry application.

## Author contribution

Qiu Zhong contributed to data analysis, interpretation of results, and validation of experimental findings. F. Raziq participated in experiment execution, data collection and analysis, interpretation of results, and manuscript drafting. S. Rahman contributed to study design, data collection and analysis, interpretation of results, and manuscript drafting. S. Khan conceived and designed the study, supervised the research, and critically revised the manuscript. G. Abbas participated in experiment execution, data collection, interpretation of results, and manuscript review. M. T. Khan contributed to data analysis, interpretation and validation of results, and critical revision of the manuscript. R. Akhtar participated in experimental work, contributed to data interpretation, and reviewed the manuscript. E. Bughio contributed to experimental methodology development, data analysis, interpretation of results, and critical revision of the manuscript. U. Farooq participated in experimental work, contributed to data interpretation, and reviewed the manuscript. Yue Yu assisted with experimental activities, contributed to interpretation of findings, and reviewed the manuscript. Z. M. Iqbal contributed to methodological development, data analysis, interpretation of results, and critical revision of the manuscript. Dalia Fouad contributed to interpretation and validation of findings and critically reviewed the manuscript. Yuwen Han contributed to interpretation of results, critically revised the manuscript, and approved the final version for publication. All authors reviewed, approved, and agreed to the published version of the manuscript.

## Disclosures

No conflict of interests was reported by the authors regarding the publication of this research article.
